# The Effect of Synbiotic Consumption on Serum NTproBNP, hsCRP and Blood Pressure in Patients With Chronic Heart Failure: A Randomized, Triple-Blind, Controlled Trial

**DOI:** 10.3389/fnut.2021.822498

**Published:** 2022-04-13

**Authors:** Shakiba Shoaei Matin, Farzad Shidfar, Nasim Naderi, Ahmad Amin, Fatemeh Sadat Hosseini-Baharanchi, Afsaneh Dehnad

**Affiliations:** ^1^Department of Nutrition, School of Public Health, Iran University of Medical Sciences, Tehran, Iran; ^2^Rajaie Cardiovascular, Medical and Research Center, Iran University of Medical Sciences, Tehran, Iran; ^3^Department of Biostatistics, Minimally Invasive Surgery Research Center, School of Public Health, Iran University of Medical Sciences, Tehran, Iran; ^4^Department of Medical Education, Center for Educational Research in Medical Sciences (CERMS), School of Health Management and Information Sciences, Iran University of Medical Sciences, Tehran, Iran

**Keywords:** synbiotic, heart failure, NT-proBNP, blood pressure, hs-CRP

## Abstract

**Background:**

In recent years, there has been a positive attitude toward gut microbiota and its effect on cardiovascular diseases, including heart failure.

**Objective:**

The purpose of this study was to evaluate the effect of synbiotics on left ventricular hypertrophy by measuring NT-proBNP, and their effect on blood pressure and hsCRP as an inflammatory biomarker in patients with chronic heart failure.

**Design:**

In this triple-blind randomized clinical trial, 90 eligible patients were included in the study. They were randomly assigned to receive one capsule (500 mg) of synbiotics or placebo per day for 10 weeks. NTproBNP, hsCRP and blood pressure were measured at the beginning and end of the study. Statistical analysis was performed on 80 patients by using SPSS 24, and *p* < 0.05 as statistically significant.

**Result:**

At the end of the study, the level of NT-proBNP decreased significantly in the synbiotic group compared to the placebo group (r = −256.55; *P* = 0.04). However, hsCRP increased in both groups as compared to the beginning of the study, but only in the placebo group the increase in hsCRP was significant (*P* = 0.01). The results showed that the changes in hs-CRP was not significant between the two groups. No statistically significant differences were observed in systolic and diastolic blood pressure between the two groups at the end of the intervention.

**Conclusion:**

Synbiotics have favorable effect on cardiac hypertrophy index (NT-proBNP). Although the inflammatory factor increased in both groups, the significant increase in hsCRP in the placebo group could indicate the beneficial effects of synbiotics on the inflammatory status of these patients.

**Clinical Trial Registration:**

https://en.irct.ir/user/trial/42905/view, identifier: IRCT20091114002709N52.

## Introduction

Chronic heart failure (CHF) is a complex clinical syndrome which is characterized by a structural or functional disorder in the heart leading to the failure to pump blood within physiological pressure levels (1). The prevalence of HF is 1–2% of the adult population in developed countries, which is growing to more than 10% among people over 70 years of age (2). It is predicted that the disease will increase further to nearly 50% by 2030, leading to an economic and social challenge in the future. A variety of pathologies, which can appear as biomarkers in the bloodstream, are attributed to heart failure. B-type natriuretic peptide (BNP) and N-terminal (NT)-pro hormone BNP (Nt-proBNP) are the most important circulatory biomarkers for the diagnosis, prognosis and monitoring of heart failure (3). BNP is a hormone secreted by the heart, and NTproBNP is a prohormone and an inactive form of BNP secreted from the same region; both of them are released in response to changes in intracardiac pressure (4). hsCRP, as an inflammatory marker, is linked to heart failure, and it has been observed that the concentration of hsCRP in the blood increases with the severity of heart failure. Studies have also shown that inflammation can increase NTproBNP, suggesting that NTproBNP and inflammation should be evaluated together (5). High blood pressure is a major and compensable risk factor for heart failure. Among patients with chronic hypertension, structural and functional changes in the heart can lead to heart failure (6). Blood pressure management prevents asymptomatic organ damage caused by hypertension, which can lead to heart failure, as well as preventing further progression of the disease (7).

In recent years, there has been a positive attitude toward gut microbiota and its effect on cardiovascular diseases, including heart failure. Several small cohort studies have shown changes in the intestinal microbial population in patients with HF (8). Changes in the composition of the gut microbiota called dysbiosis can cause systemic inflammation which is involved in the pathophysiology of HF (9). Intestinal microbiota dysbiosis can also lead to increased trimethylamine-N-oxide (TMAO) which is an important gut-microbiota metabolites generated from dietary choline, betaine, and L-carnitine (8). Elevated TMAO levels are associated with a higher level of NTproBNP, more severe left ventricular dysfunction, left ventricular dilatation, and poor outcomes in patients with heart failure (9–11). Modification of intestinal microbiota with probiotics may be a new way to prevent and treat cardiac remodeling in humans (12). Synbiotics are a combination of probiotics and prebiotics which improve the balance of intestinal microbiota by altering the microbial composition and modulating the susceptibility to cardiovascular diseases and preventing the progression to HF (8). Probiotics and synbiotic therapy can reduce inflammatory factors such as hsCRP. Reduction of inflammatory factors by probiotics is likely through improving intestinal barrier functions, reducing pro-inflammatory stimuli such as lipopolysaccharides (LPSs), producing short-chain fatty acids (SCFAs), increasing the production of anti-inflammatory cytokines (IL10), and decreasing pro-inflammatory cytokines (tumor necrosis factor-α (TNF-α) and interleukin-6) (13, 14). People who experience hypotension due to synbiotic therapy show increased production of short-chain fatty acids (SCFA) which indicates the modulation of blood pressure and hsCRP after synbiotic intervention depends on the increased capacity of short-chain fatty acids production in the intestine (15). Due to the association between hypertrophy and HF and based on the hypothesis that synbiotics can play a positive role in the treatment of heart failure by reducing inflammation and NT-proBNP values, and the lack of a study to directly assessing the effect of synbiotics on the specific biomarker of heart failure) NT-proBNP (, we decided to evaluate the effect of synbiotics on left ventricular hypertrophy by measuring NT-proBNP and its effect on blood pressure and hsCRP as inflammatory biomarker in the patients.

## Method

### Participants

Participants were selected from patients with heart failure who were referred to Shahid Rajaie hospital, Tehran, Iran between October 2019 and August 2020. All items included in the checklist of Extending the CONSORT Statement to Randomized Trials of Non-pharmacologic Treatment were fulfilled. Patients with heart failure, aged between 30 and 70 years with LVEF <40 (left ventricular ejection fraction), who had been approved by a cardiologist and had received standard treatments for heart failure for at least 3 months and the medications had reached the maximum tolerable and constant dose, and those who were in one of I–III stages of the NYHA (New York Heart Association (classifications were included in the study.

As for the exclusion criteria in this study, we excluded patients with chronic and acute liver disorders (hepatitis B, C, etc.), diabetes, thyroid disorders, severe renal impairment (creatinine ≥300 mmol/L), lung diseases, inflammatory and autoimmune diseases, cancer, and acute infections. We also excluded those patients taking any nutritional supplements within the past 2 months, corticosteroids within the past 4 weeks, antibiotics within the past 3 months, as well as taking anti-inflammatory drugs except for low-dose aspirin (80 mg daily). Meanwhile, patients with a history of gastrointestinal surgery, rheumatic heart disease, and prosthetic heart valve were excluded from this study. Further exclusion criteria included smoking, pregnancy and lactation, insulin injection, BMI>30 kg/m^2^, consuming <80% of supplements during the study period, as well as experiencing changes in the type and dose of heart failure medication. Also, in order to follow our strict Code of Ethics, we excluded those who were reluctant or mentally unprepared to participate in this study.

### Ethical Aspects

This study was approved by the ethics committee of Iran University of Medical Sciences (approval no. IR.IUMS.REC.1398.675) and was registered in the Iranian Clinical Trials Register (registration no. IRCT20091114002709N52). Informed consent forms and signatures were received from all the participants before any intervention. Participants were told that they could withdraw from the study at any stage of the project if they did not wish to continue cooperating with the project, and were assured that their information would remain confidential.

### Study Design

This study was performed as a randomized, triple-blind, placebo-controlled, parallel- group clinical trial in patients with heart failure. Participants entered the run-in period and did not consume any probiotic products and yogurt, for 2 weeks prior to the beginning of the study. Patients were asked not to consume yogurt, any probiotic products and any nutritional supplement during the study. They were also asked to maintain their regular life including physical activity and diet. Participants were randomized into two groups. experimental group (*n* = 45) received one synbiotic capsule (each capsule = 0.5g) and control group (*n* = 45) received one placebo capsule (each capsule = 0.5 g) per day for 10 weeks. They were/told to keep the supplements in the refrigerator. Patient compliance s was checked by telephone calls and they were requested to bring their capsule box in the last visit.

### Sample Size

A similar study to evaluate the effect of synbiotics on NT-proBNP had not been performed. Therefore, the sample size was calculated and reported according to two secondary outcomes and the highest number (39 samples according to diastolic blood pressure) was determined as the sample size (16, 17). A sample size of 45 patients in each of the two study groups, with a dropout rate of up to 15%, a power of 80%, and 5% probability of type one error was planned to detect a significant reduction in diastolic blood pressure in the symbiotic group, compared with the placebo group after 6 weeks, at the end of the study.

### Randomization

All patients were distributed between the two groups by quadratic-blocks randomization and a random list was prepared by statistical software. Randomization and blinding were performed to maintain concealment in the study.

### Intervention

Participants were randomized into two groups. One group (*n* = 45) received one synbiotic capsule (0.5 g) which contained *Lactobacillus casei (*10^9^ cfu)*, Lactobacillus acidophilus (1.5* × 10^10^ cfu)*, Lactobacillus rhamnosus (3.5* × 10^9^ cfu)*, Lactobacillus bulgaricus (2.5* × 10^8^ cfu)*, Bifidobacterium breve (*10^10^ cfu)*, Bifidobacterium longum (5* × 10^8^ cfu)*, Streptotus thermophilus (1.5* × 10^8^ cfu), and excipients included fructooligosaccharide (38.5 mg), lactose, magnesium stearate, talc, and silicon dioxide. The other group (*n* = 45) received one placebo capsule (0.5 g) containing lactose, magnesium stearate, talc and silicon dioxide per day for 10 weeks. In a study that examined the effect of the probiotic Saccharomyces bulardii on patients with heart failure, it was found that the proposed treatment with probiotic is safe and well-tolerated and there are no reports of side effects in patients (16). The synbiotic and placebo capsules were the same in appearance, odor, weight and packaging, and only the code on the package (A or B) was different. Patient were asked to store synbiotic capsules in the refrigerator.

### Assessments

#### Anthropometric Measures

Participants' weight with minimum coverage and without shoes was measured by using Seca scales with an accuracy of 100 grams and the standing height was measured without shoes by a gauge with an accuracy of 0.5 cm at the beginning and end of the study. BMI was calculated by using the formula (BMI = weight/height^2^). For most adults, an ideal BMI is in the 18.5–24.9 (kg/m^2^) range. BMI scores of 25–29.9 (kg/m^2^) are considered overweight. Normal and overweight BMI were included in the present study.

#### Dietary Intakes and Physical Activity

Dietary information was collected at the beginning of the study and at the end of the 10^th^ week by using 24-h food recall for 3 days (2 non-holidays and 1 weekend), and the usual intake of energy, macronutrient and micronutrient was calculated by Nutritionist IV software. The amount of physical activity was assessed by using the short form of the International Physical Activity Questionnaire (IPAQ) (18) at the beginning and end of study. The metabolic equivalent was defined as the sum of all physical activities per minute during the week.

#### Biochemical Assessment

To measure blood factors, venous blood samples were taken from patients at the beginning of the study and at the end of the 10^th^ week after 10–12 h of fasting by a laboratory expert. Blood samples were stored at −80°C during the study. The concentration of NTproBNP was measured by using ELISA method (Crystalday kit, China), and hsCRP by immunoturbidometery method (Pars Azmoun, Iran).

#### Blood Pressure

Systolic and diastolic blood pressure was measured via the right arm in a sitting position after 10 min of rest in the morning by a digital sphygmomanometer at the beginning and end of the study. People who entered the study had controlled blood pressure and were under the supervision of a doctor, and we did not include people who had uncontrolled high blood pressure (>14/8 mmHg).

#### Statistical Analysis

In this study after collecting data, demographic and descriptive information as well as the relationship between variables were examined using statistical methods, all analyzes were performed with SPSS-24 software. Quantitative data were described as mean ± SD and median. qualitative variables were also reported as frequency in percentage. Kolmogorov-Smirnov test and other indicators of normality were used to check the normality of the data. Value for between-group comparison of qualitative data was performed by using Chi square or Fisher's exact test. Intergroup comparison of quantitative data was performed by using independent-samples *T* test and intragroup comparison was performed using paired-samples *T* test. ANCOVA method was used to evaluate the effect of the intervention performed after adjusting based on the baseline values of the outcomes and BMI as covariates.

## Results

**Figure 1:**
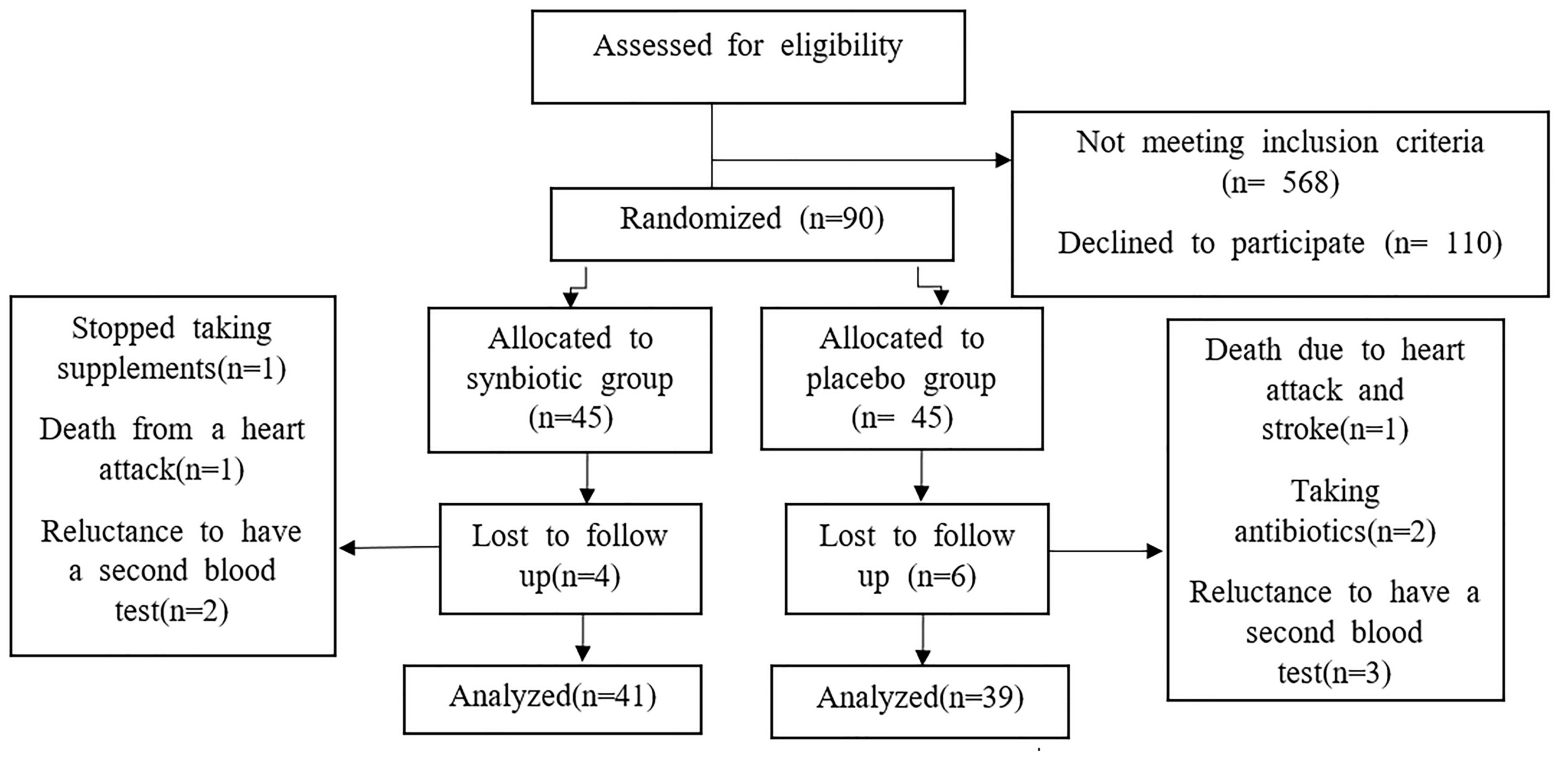
Shows the study consort flow chart.

**Figure 2:**
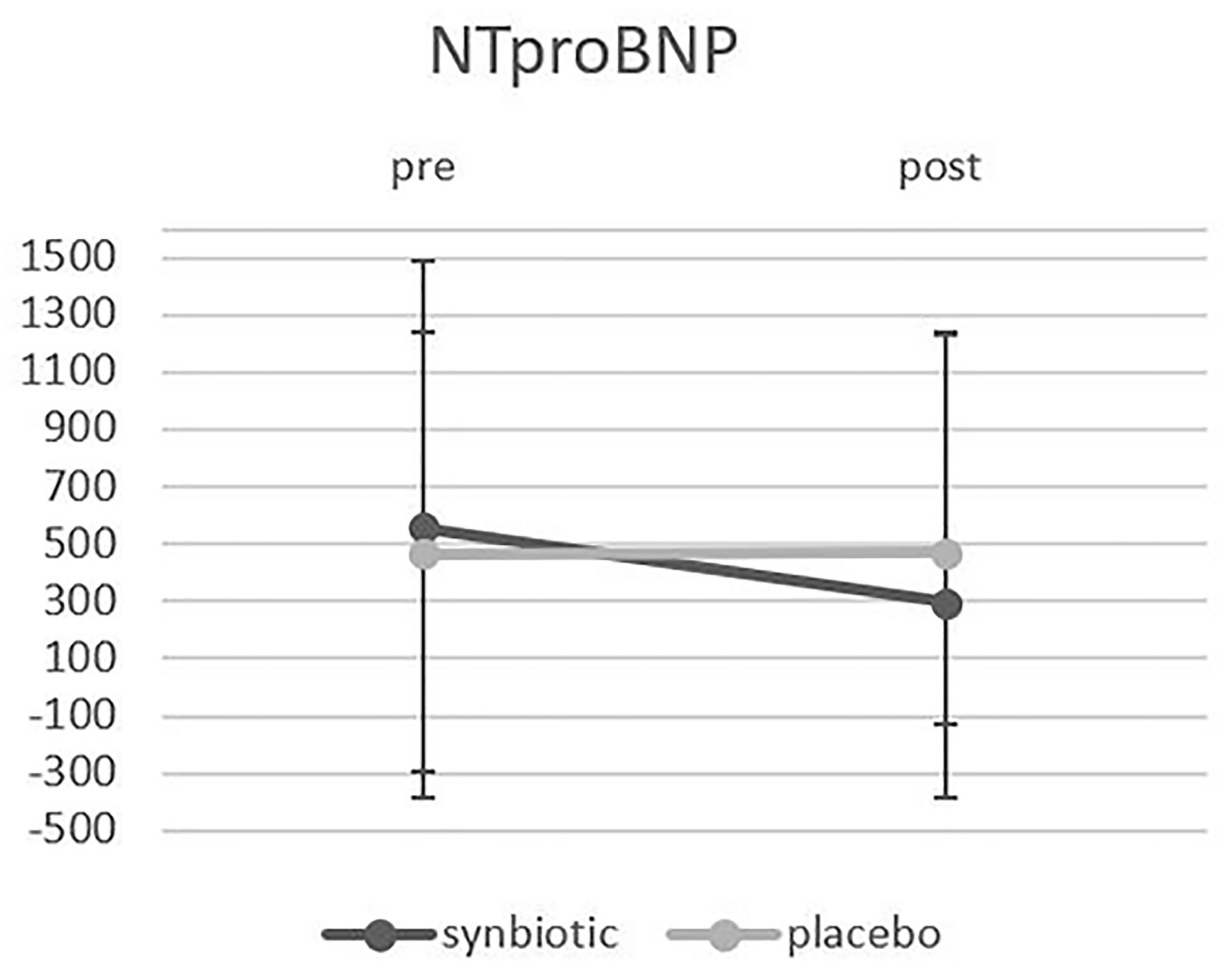
Shows the Comparison of NT-proBNP changes between the two groups before and after the intervention.

**Figure 3:**
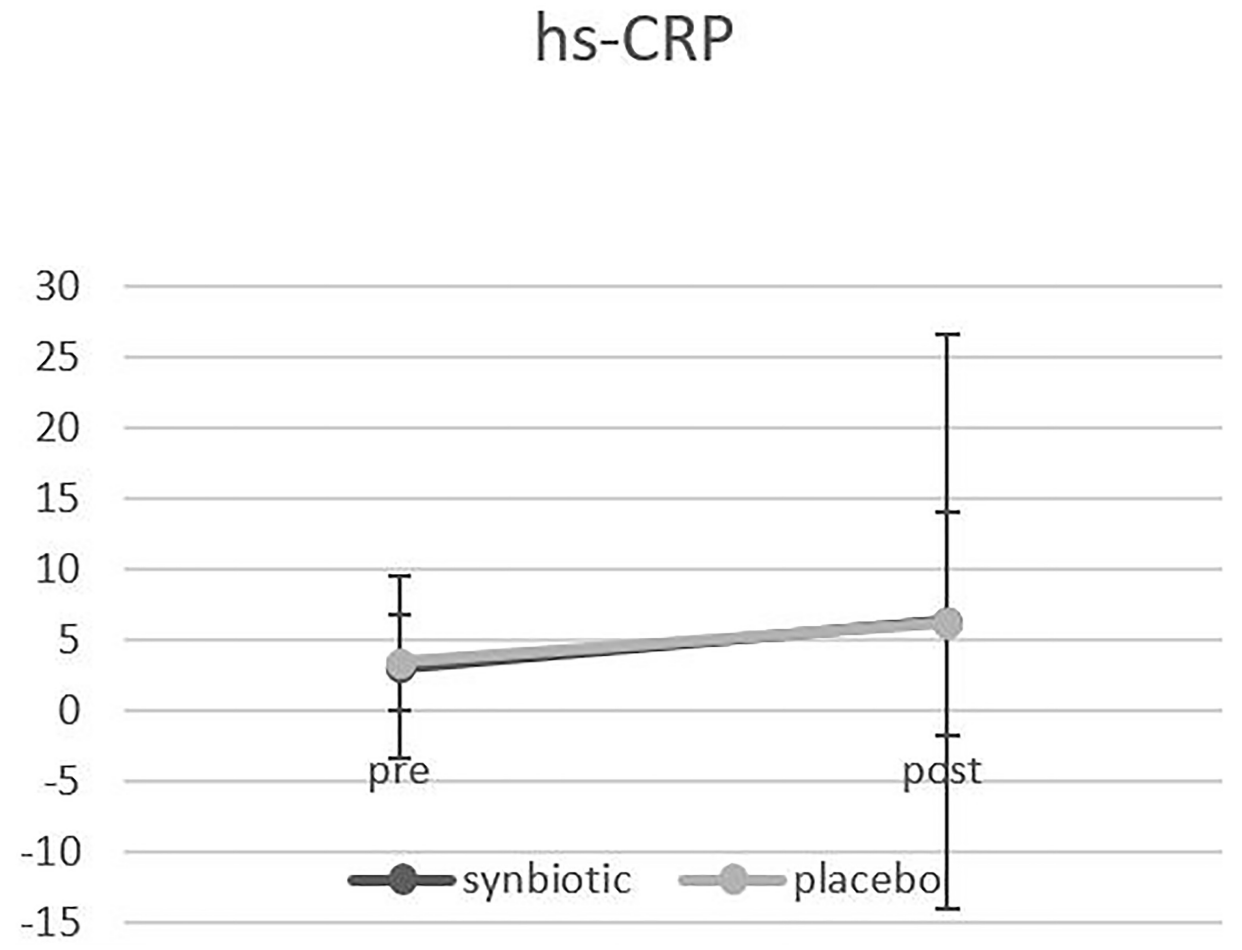
Shows the Comparison of hs-CRP changes between the two groups before and after the intervention.

From the participants enrolled in this clinical trial, 90 patients who met the inclusion criteria were randomly allocated to the synbiotic and the placebo group. During the study, four patients from the synbiotic group and six patients from the placebo group were excluded, and 80 patients completed the study (Figure 1). There were no significant differences between the two groups in terms of gender, marriage, age, weight, Body Mass Index (BMI), duration of heart failure and ejection fraction. The findings regarding disease-related variables and medications used by participants also showed that there were no significant differences between the two groups in terms of disease-related variables, medications and smoking (Tables 1, 2). There was no significant difference in the intake of macronutrients and micronutrients between the two groups (Supplementary Table 1). According to the results, the level of NT-proBNP in the synbiotic group decreased significantly and the number of changes between the two groups at the end of the study was significant (*p* = 0.04) (Figure 2). There was no statistically significant difference in serum hs-CRP levels between the two groups. In fact, hs-CRP levels increased in both synbiotic and placebo groups, but it should be noted that this increase was significant only in the placebo group (*p* = 0.01) (Figure 3, Table 3). We observed a decrease in systolic and diastolic blood pressure in the synbiotic group and an increase in systolic and diastolic blood pressure in the control group at the end of the study compared to the initial values; however, these changes were not statistically significant (Table 4).

**Table 1:** Individual characteristics of study participants.

**Variable**	**Groups**		***p*-value**
	**Synbiotic**	**Placebo**	
	**(*n* = 41)**	**(*n* = 39)**	
**Age (years)**	50.54 ± 11.4	50.1 ± 10.56	0.86
**Sex**, ***n*** **(%)**			
Female	9 (22)	14 (35.9)	0.16
Male	32 (78)	25 (64.1)	
**LVEF**	26.20 ± 6.82	24.23 ± 8.7	0.26
**NYHA classification**, ***n*** **(%)**			
Class 1	18 (43.9)	15 (38.5)	0.72
Class 2	19 (46.3)	18 (46.2)	
Class 3	4 (9.8)	6 (15.4)	
**Stent**, ***n*** **(%)**			1
Yes	4 (9.8 )	3 (7.7)	
No	37 (90.2)	36 (92.3)	
**Pacemaker**, ***n*** **(%)**			
Yes	3 (7.3)	6 (15.4)	0.3
No	38 (92.7)	33 (84.6)	
**Duration of the disease (month)**	4,615 ± 42.93	43.36 ± 35.51	0.75
**Taking statins**, ***n*** **(%)**			
Yes	12 (29.3)	9 (23.1)	0.52
No	29 (70.7)	30 (76.9)	
**Taking MRAs**, ***n*** **(%)**			
Yes	32 (78)	36 (92.3)	0.07
No	9 (22)	3 (7.7)	
**Taking diuretics**, ***n*** **(%)**			
Yes	27 (65.9)	30 (76.9)	0.27
No	14 (34.1)	9 (23.1)	
**Taking beta-blockers**, ***n*** **(%)**			
Yes	34(82.9)	36(92.3)	0.31
/No	7(17.1)	3(7.7)	
**/Digoxin**, ***n*** **(%)**			
Yes	5(12.2)	6(15.4)	0.67
No	36(87.8)	33(84.6)	
**ACEI.ARBs**, ***n*** **(%)**			
Yes	36(87.8)	31(79.5)	0.31
No	5(12.2)	8(20.5)	
**Taking anticoagulants**, ***n*** **(%)**			
Yes	21(51.2)	15 (38.5)	0.25
No	20 (48.8)	24 (61.5)	
**Taking vasodilators**, ***n*** **(%)**			
Yes	1 (2.4)	2 (5.1)	0.61
No	40 (97.6)	37 (94.9)	
**Smoking**, ***n*** **(%)**			
Yes	7 (17.1)	4 (10.3)	0.37
No	34 (82.9)	35 (89.7)	

*Data are presented as mean (SD) for quantitative and frequency (%) for qualitative variables. Value for between-group comparison of qualitative data was performed by using Chi square or Fisher's exact test and between-group comparison of parametric quantitative data was performed by using independent sample t-test. ACEI, Angiotensin-Converting Enzyme Inhibitors; ARBs, Angiotensin II type I receptor blockers; LVEF, left ventricular ejection fraction; MRAs, Mineralocorticoid Receptor Antagonists; NYHA, New York Heart Association*.

**Table 2:** Anthropometric indices at baseline, at the end of the study and changes between the synbiotic and placebo groups.

**Variables**		**Synbiotic (*n* = 41)**	**Placebo (*n* = 39)**	***p*-value**
Weight (kg)	Baseline	75.14 ± 12.60	77.47 ± 13.72	0.43
	10^th^ week	73.64 ± 11.62	78.64 ± 13.82	0.08
	Change	−1.50 ± 1.71	1.17 ± 1.69	<001/0
	*p*-value	<001/0	<001/0	
BMI (kg/m^2^)	Baseline	25.51 ± 3.77	26.53 ± 2.87	0.18
	10^th^ week	25.10 ± 3.42	27.06 ± 2.97	0.008
	Change	−0.41 ± 0.81	0.53 ± 0.66	<001/0
	*p*-value	0.002	<001/0	
Physical activity (MET-minutes/week)	Baseline	184.15 ± 190.1	216.21 ± 252.89	0.52
	10^th^ week	118.22 ± 126	155.49 ± 192.67	0.3
	Change	−65.92 ± 125.45	−60.71 ± 228.25	0.89
	*p*-value	0.002	0.1	

*Data are presented as mean (SD). Intergroup comparison of quantitative data was performed by using independent-samples T test and intragroup comparison was performed using paired-samples T test*.

**Table 3:** Biochemical measurement at baseline, at the end of the study and changes between the synbiotic and placebo groups.

**Variable**		**Synbiotic (*n* = 41)**	**Placebo (*n* = 39)**	***p*-value**	**Adjusted *p*-value 1**	**Adjusted *p*-value 2**
NT-proBNP (pg/ml)	Baseline	555.20 ± 937.04	472.19 ± 767.89	0.66	0.03	0.04
	10^th^ week	298.64 ± 430.71	474.18 ± 858.31	0.24		
	Change	−256.55 ± 697.95	1.98 ± 416.91	0.04		
	*p*-value	0.02	97/0		0.47	0.7
hsCRP(mg/l)	Baseline	3.11 ± 6.46	3.48 ± 3.41	0.74		
	10^th^ week	6.4 ± 20.31	6.22 ± 7.88	0.95		
	Change	3.28 ± 14.3	2.73 ± 6.52	0.82		
	*p*-value	0.14	0.01			

*Data are presented as mean (SD). Intergroup comparison of quantitative data was performed by using independent-samples T test and intragroup comparison was performed by using paired-samples T test. Adjusted p-value 1 was performed by using ANCOVA test on baseline values. Adjusted p-value 2 was performed by using ANCOVA test on baseline values and BMI. hs-CRP, high-sensitivity C-reactive protein; NT-proBNP, N-Terminal pro-brain natriuretic peptid*.

**Table 4:** Findings related to blood pressure at baseline, at the end of the study and changes between the synbiotic and placebo groups.

**Variable**		**Synbiotic (*n* = 41)**	**Placebo (*n* = 39)**	***p*-value**	**Adjusted *p*-value 1**	**Adjusted *p*-value 2**
Systolic blood pressure (mmHg)	Baseline	11.47 (1.37)	11.17 (2.00)	0.44	0.75	0.87
	Week 10	11.3 (1.21)	11.21 (1.39)	0.76		
	Change	−0.17 (0.84)	0.03 (1.58)	0.46		
	*p*-value	0.2	0.88		0.92	0.55
Diastolic blood pressure (mmHg)	Baseline	7.43 (1.07)	7.28 (1.29)	0.55		
	Week 10	7.39 (0.81)	7.33 (0.89)	0.76		
	Change	−0.04 (0.73)	0.05 (1.09)	0.63		
	*p*-value	0.67	0.77			

*Data are presented as mean (SD). Intergroup comparison of quantitative data was performed by using independent-samples T test and intragroup comparison was performed by using paired-samples T test. Adjusted p-value 1 was performed by using ANCOVA test on baseline values. Adjusted p-value 2 was performed by using ANCOVA test on baseline values and BMI*.

## Discussion

The findings of the present study show that synbiotics have some effects on the serum levels of NTproBNP in patients with CHF, while they have no significant effects on hsCRP and blood pressure. To our knowledge, this is the first clinical trial which has been performed on the effect of synbiotics on NT-proBNP factor in patients with heart failure. Several clinical trial studies have been performed on the NT-proBNP factor which have shown consistent results with the current study (19–21). Pei-Pei Lin et al. examined the inhibitory effect of probiotic-fermented purple sweet potato yogurt (PSPY) on cardiac hypertrophy in 22 6-week-old male mice. The mice were divided into four groups: the control group receiving hypertension (SHR), the second group receiving captopril, the third group receiving 10% PSPY, the fourth group receiving 100% PSPY, and the negative control group. After 8 weeks ppathological hypertrophy markers such as left ventricular BNP was higher in the SHR group than the negative control group, and it was lower in the 10,100% PSPY groups than the control group (SHR). The mechanism mentioned for the anti-hypertrophic effect was through the reduction of IL-6 and IGF-II (22). Also, another study which was conducted to investigate the effect of short-term use of resveratrol in combination with calcium fructoborate on NT-proBNP, lipid profile and inflammatory markers in patients with stable angina revealed that 2-month use of resveratrol alone with calcium fructoborate resulted in a significant reduction in NT-proBNP, which is consistent with the results of our study (23).

The results of some studies on serum NT-proBNP levels are inconsistent with the findings of the current study. Wujec-Makarewicz et al. reported that there were no changes on NT-proBNP levels, after 30 weeks of omega-3 fatty acid intake in a dose of 1 g per day in heart failure patients. They reported a limited sample size study and suggested that further research with higher sample sizes was needed to better understand the relationship between patient diet and the effectiveness of omega-3 supplements (24). Another study showed that taking folic acid supplementation for 8 weeks had no effect on NT-proBNP in healthy individuals with NT-proBNP <40 ng/l and could be effective, resulting to a decreased NT-proBNP in individuals with NT- proBNP > 40 ng/l. It has also been mentioned that relatively small treatment groups and large interpersonal differences in NT-proBNP levels are other factors that may prevent a positive outcome (25). In a study comparing the consumption of probiotic yogurt and regular yogurt by patients with chronic heart failure, it was found that after 10 weeks of consumption of probiotic yogurt and regular yogurt, no changes in NT-proBNP levels were observed between the two groups. A possible reason for that finding could be the low baseline level of NT-proBNP at the beginning of the study (26).

Dysbiosis and alteration of intestinal microbes can impair endothelial function by increasing reactive oxygen species (ROS), increasing lipopolysaccharides, decreasing SCFAs in the intestine, and reducing Nitric Oxide (NO) release in the endothelium, leading to chronic hypertension, which increases the severity of cardiac overload. As a result, we would observe an increase in NT-proBNP levels, which is one of the most important indicators of increased cardiac overload (27). Probiotic bacteria such as lactobacilli and bifidobacteria play a direct role in NO production, while pathogenic bacteria of the intestinal flora such as E. coli consume NO (28). Increased NO plays a role in lowering blood pressure, and the anti-proliferative effect of NO can prevent cardiac hypertrophy (29). On the other hand, the results of a recent meta-analysis study show that the use of synbiotics in comparison with probiotics can cause a significant increase in NO levels. In fact, the effect of synbiotics on increasing NO levels is greater than probiotics, and this difference is probably due to the higher production of SCFAs such as butyrate by prebiotics (inulin and fructooligosaccharides) in the gut compared to probiotics, which leads to a greater increase in NO (28). Therefore, it is possible that the use of prebiotics along with probiotics might have a greater effect on increasing NO levels and subsequently lowering serum NTproBNP levels as compared to using probiotics alone. Dysbiosis also leads to an increase in dangerous metabolites of bacteria such as TMAOs and a decrease in beneficial metabolites such as SCFAs, which ultimately affects cardiac physiology. Recent studies have shown that probiotics reduce TMAO levels, and their protective effects on the heart may be partially achieved by lowering circulating TMAO. In addition, the TMAO level is directly related to the NYHA classification and the NT-proBNP level (30).

In the present study, there was no statistically significant difference in serum hs-CRP levels between the two groups at the end of the study. In fact, hs-CRP levels increased in both synbiotic and placebo groups; however, it should be noted that this increase was significant in the placebo group (*p* = 0/01). To the best of our knowledge, no study has examined the effect of synbiotics on hs-CRP levels in patients with heart failure. Ghanei et al. reported that the daily consumption of two probiotic supplements (*acidophilus, L. plantarum, L. fermentum, L. gaseri* at 10^9^ CFU / gr) for 12 weeks in 60 patients with polycystic ovary syndrome showed a significant decrease in serum hs-CRP levels within both groups at the end of the study, but in line with our study, no difference was observed between the two groups (31). Similarly, in another study performed on patients with type two diabetes, the effects of daily consumption of two probiotic supplements *(L.acidophilus, L. bulgaricus, L. bifidum, L. casei*) for 6 weeks were investigated. At the end of that study, consistent with the results of current study, serum hs-CRP levels increased in both groups, although it was not statistically significant (32). In another study performed on patients with type two diabetes, it was observed that daily consumption of two probiotic sachets (*B. bifidum W23, B. lactis W52, L. acidophilus W37, L. brevis W63, L. casei W56, L. Salivarius W24, Lactococcus lactis W19, and L. lactis W58* (2.5 × 10^9^ CFU / gr) for 6 months led to a significant decrease in CRP levels in the intervention group compared to baseline values but no significant difference was observed between the groups. It seems that the lack of effect between the groups was due to the high level of endotoxin and basal adipokine in the probiotic group compared to the placebo group (33). Studies on the effects of omega-3 on the hsCRP factor have shown no effect and the reason was the low initial level of hsCRP (34). In the present study, the initial level of hsCRP was low (3.11 ± 6.46 in the synbiotic group compare to 3.48 ± 3.41 in the placebo group), which could be the reason for no significant effect of synbiotics on hsCRP. On the other hand, in a study on the inflammatory status of diabetic patients after 8 weeks of taking synbiotic supplementation, a significant decrease in the levels of hsCRP, IL-6 and TNF-α was observed. The mean initial hsCRP level in that study was 4.9 ± 2.36 for the synbiotic group and 5 ± 2.31 for the placebo group, which was higher than the mean of hs-CRP in our study (35). The results of that study also shows that the duration of our intervention was sufficient to affect inflammatory factors. Increased number of pathogenic bacteria exacerbates systemic inflammation by reaching the bloodstream through a dysfunctional intestinal system; therefore, targeting the intestinal microbial composition is extremely important. Probiotic therapy may reduce inflammatory factors by repairing the epithelial barrier, modulating and enhancing the local and systemic immune response, improving intestinal barrier function, modifying the activity of natural killer cells (NK cells), modulating the NF-κB pathway, inducing T cell apoptosis, increased production of intestinal anti-inflammatory cytokines such as IL-10, reduced production of proinflammatory markers, and reduced production of SCFA (14, 36). Moreover, it is evident that increasing TMAO plasma levels and increasing intestinal permeability increase the risk of cardiovascular diseases due to chronic inflammation and endothelial dysfunction. The mechanisms by which TMAO activates inflammatory pathways are still being studied. However, the role of NF-κB proinflammatory pathway activation in triggering the inflammatory response in the presence of large amounts of TMAO is significant (37).

Although we observed a decrease in blood pressure after the intervention in the synbiotic group and an increase in blood pressure in the control group, these numbers were not statistically significant and there was no significant difference between the two groups at the end of the intervention. Similar studies have reported the same finding indicating no significant difference in systolic and diastolic blood pressure after synbiotic supplementation. In a study evaluating the blood pressure lowering effects of fermented milk by *lactobacillus helveticus*, a significant difference in systolic and diastolic blood pressure was observed between the groups. In this study, the species *Lactobacillus helveticus* was used and the reason for this choice was the production of biological peptides by the species of Lactobacillus, which have a similar activity to the angiotensin converting enzyme inhibitor and are even more effective than these kind of medications. Another mechanism mentioned for the antihypertensive effect of fermented milk is the calcium content of milk (38). In a review and meta-analysis study on the effect of probiotics on blood pressure, conducted in 2020, the beneficial effects of probiotics on blood pressure were further demonstrated by dairy products in people with blood pressure >130.80. It should be noted that the average blood pressure in our study was 110.70 (39).

The key strength of the present is that this is the first study evaluating the effect of synbiotics in patients with heart failure. Moreover, the patients were selected in such a way that there was no significant difference between the characteristics of the two groups before the intervention, indicating that very accurate and principled randomization was performed.

However, there are some limitations to the present study. We did not examine the intestinal microbiome at the beginning, during and after taking synbiotics due to financial limitations.

## Conclusion

Overall, in this study, synbiotic consumption in patients with chronic heart failure for 10 weeks resulted in a significant decrease in NT-proBNP levels compared to the control group but it had no effect on the inflammatory factor hs-CRP, and systolic and diastolic blood pressure. Further studies are suggested to determine the mechanisms of action of probiotics by measuring the levels of SCFAs and TMAOs.

## Data Availability Statement

The raw data supporting the conclusions of this article will be made available by the authors, without undue reservation.

## Ethics Statement

The studies involving human participants were reviewed and approved by https://www.irct.ir/ Iran University of Medical Sciences, Tehran, Iran. The patients/participants provided their written informed consent to participate in this study.

## Author Contributions

FS, SS, and NN designed the research. SS conducted the research and wrote the paper. NN and AA heart failure specialists who made final approval of patients to enter the study. FH-B analyzed the data. FS had primary responsibility for final content. AD English specialist who has translated the language of the article into Native English. All authors reviewed the manuscript and approved it prior to submission.

## Funding

This RCT was funded by Iran University of Medical Sciences, Tehran, Iran.

## Conflict of Interest

The authors declare that the research was conducted in the absence of any commercial or financial relationships that could be construed as a potential conflict of interest.

## Publisher's Note

All claims expressed in this article are solely those of the authors and do not necessarily represent those of their affiliated organizations, or those of the publisher, the editors and the reviewers. Any product that may be evaluated in this article, or claim that may be made by its manufacturer, is not guaranteed or endorsed by the publisher.

## Abbreviations

ACEI: Angiotensin-Converting Enzyme Inhibitors
ARBs: Angiotensin II type I receptor blockers
BMI: Body-mass index
CFU: colony-forming unit
CHF: Chronic Heart Failure
EF: Ejection fraction
HF: Heart failure
HsCRP: High Sensitivity C-Reactive Protein
IL-10: Interleukin 10
LPS: LipoPolySaccharide
LVEF: Left ventricular ejection fraction
MRAs: Mineralocorticoid/aldosterone receptor antagonists
NF-κB: Nuclear factor-Kb
NK cells: Natural killer cells
NTproBNP: N-Terminal pro-brain natriuretic peptide
NYHA: New York Heart Association
SCFAs: Short-chain fatty acids
TMAO: Trimethylamine N-oxide
TNF-α: Tumor necrosis factor-α.
