# Supplementary material for: The Effect of Synbiotic Consumption on Serum NTproBNP, hsCRP and Blood Pressure in Patients With Chronic Heart Failure: A Randomized, Triple-Blind, Controlled Trial
Source: Front Nutr. 2022 Apr 13;8:822498. doi: 10.3389/fnut.2021.822498 (PMC9043653; doi:10.3389/fnut.2021.822498)
Supplement: Supplementary file 1 [file Table_1.pdf]

Supplementary Table 1. Findings related to the evaluation of nutrients received from the diet at baseline, at the end of the study and changes between the synbiotic and placebo groups

Variables		Synbiotic (n=41)	Placebo (n=39)	p-value
Energy (Kcal/d)	Baseline	2285/51 (434/59)	2454/11 (500/67)	0/11
	10th week	2410/52 (448/84)	2561/11 (409/64)	0/12
	Change	125/00 (258/65)	107/00 (379/41)	0/80
	p-value	0/004	0/08	
carbohydrate (gr/d)	Baseline	365/49 (81/25)	391/13 (87/21)	0/17
	10th week	373/26 (76/02)	402/48 (73/17)	0/08
	Change	7/76 (60/90)	11/34 (75/20)	0/81
	p-value	0/41	0/35	
Fiber (gr/d)	Baseline	17/32 (5/42)	19/11 (5/59)	0/14
	10th week	18/04 (3/75)	18/55 (5/33)	0/62
	Change	-0/56 (5/56)	0/71 (4/41)	0/25
	p-value	0/52	0/29	
Protein (gr/d)	Baseline	88/32 (23/92)	91/80 (20/48)	0/48
	10th week	90/10 (22/57)	95/15 (20/10)	0/29
	Change	1/77 (21/35)	3/34 (20/02)	0/73
	p-value	0/59	0/30	
Fat (gr/d)	Baseline	53/77 (16/79)	60/63 (17/30)	0/07
	10th week	63/92 (16/39)	65/72 (14/12)	0/60
	Change	10/14 (16/33)	5/08 (16/28)	0/16
	p-value	<0/001	0/05	
sodium(mg/d)	Baseline	1431/22 (640/65)	1468/29 (651/43)	0/79
	10th week	1717/15 (769/17)	1603/46 (673/35)	0/48
	Change	285/93 (888/99)	135/17 (759/95)	0/41
	p-value	0/04	0/27	
Potassium (mg/d)	Baseline	3023/87 (1232/33)	3125/16 (903/46)	0/67
	10th week	3180/60 (671/03)	3229/18 (741/40)	0/75

	Change	156/72 (1214/05)	104/02 (929/34)	0/82
	p-value	0/41	0/48	
Magnesium (mg/d)	Baseline	292/24 (151/83)	279/89 (78/07)	0/65
	10th week	283/55 (61/97)	292/65 (59/06)	0/50
	Change	-8/69 (152/07)	12/75 (72/54)	0/42
	p-value	0/71	0/27	
Calcium (mg/d)	Baseline	(279/09) 785/66	793/05 (239/32)	0/89
	10th week	798/51 (209/40)	826/23 (212/96)	0/55
	Change	12/84 (315/80)	33/18 (203/59)	0/73
	p-value	0/79	0/31	
Selenium (mg/d)	Baseline	0/14 (0/06)	0/16 (0/06)	0/10
	10th week	0/15 (0/05)	0/16 (0/04)	0/40
	Change	0/005 (0/08)	-0/007 (0/07)	0/43
	p-value	0/65	0/51	
Iron (mg/d)	Baseline	23/17 (10/00)	21/52 (5/70)	0/37
	10th week	21/01 (4/60)	22/56 (5/71)	0/18
	Change	-2/16 (9/59)	1/04 (5/26)	0/07
	p-value	0/15	0/22	
Vitamin E (mg/d)	Baseline	6/10 (3/30)	6/84 (3/62)	0/34
	10th week	6/69 (4/31)	7/85 (4/61)	0/24
	Change	0/58 (5/25)	1/01 (5/64)	0/73
	p-value	0/47	0/27	
Vitamin D (mg/d)	Baseline	0/85 (1/04)	0/96 (1/11)	0/64
	10th week	0/67 (0/81)	0/95 (0/90)	0/14
	Change	-0/18 (1/38)	-0/01 (1/30)	0/57
	p-value	0/40	0/95	
Vitamin C (mg/d)	Baseline	147/63 (149/36)	136/38 (103/68)	0/69
	10th week	155/20 (113/65)	128/25 (62/56)	0/19
	Change	7/56 (176/99)	-8/13 (116/82)	0/64

p-value	0/78	0/66
